# Small-size recombinant adenoviral hexon protein fragments for the production of virus-type specific antibodies

**DOI:** 10.1186/s12985-017-0822-5

**Published:** 2017-08-18

**Authors:** Martin Pacesa, Rodinde Hendrickx, Manuela Bieri, Justin W. Flatt, Urs F. Greber, Silvio Hemmi

**Affiliations:** 1 Institute of Molecular Life Sciences, University of Zurich, CH-8057 Zurich, Switzerland; 2 Molecular Life Sciences Graduate School, Eidgenössische Technische Hochschule and University of Zurich, CH-8057 Zurich, Switzerland

**Keywords:** Adenovirus, Hexon, Protein Purification, Antibodies, Immunofluorescence, Neutralization, Virus Blocking, Immunoblotting, Electron Microscopy

## Abstract

**Background:**

Adenoviruses are common pathogens infecting animals and humans. They are classified based on serology, or genome sequence information. These methods have limitations due to lengthy procedures or lack of infectivity data. Adenoviruses are easy to produce and amenable to genetic and biochemical modifications, which makes them a powerful tool for biological studies, and clinical gene-delivery and vaccine applications. Antibodies directed against adenoviral proteins are important diagnostic tools for virus identification in vivo and in vitro, and are used to elucidate infection mechanisms, often in combination with genomic sequencing and type specific information from hyper-variable regions of structural proteins.

**Results:**

Here we describe a novel and readily useable method for cloning, expressing and purifying small fragments of hyper-variable regions 1-6 of the adenoviral hexon protein. We used these polypeptides as antigens for generating polyclonal rabbit antibodies against human adenovirus 3 (HAdV-B3), mouse adenovirus 1 (MAdV-1) and MAdV-2 hexon. In Western immunoblots with lysates from cells infected from thirteen human and three mouse viruses, these antibodies bound to homologous full-length hexon protein and revealed variable levels of cross-reactivity to heterologous hexons. Results from immuno-fluorescence and electron microscopy studies indicated that HAdV-B3 and MAdV-2 hexon antibodies recognized native forms of hexon.

**Conclusions:**

The procedure described here can in principle be applied to any adenovirus for which genome sequence information is available. It provides a basis for generating novel type-specific tools in diagnostics and research, and extends beyond the commonly used anti-viral antibodies raised against purified viruses or subviral components.

**Electronic supplementary material:**

The online version of this article (doi:10.1186/s12985-017-0822-5) contains supplementary material, which is available to authorized users.

## Background

Adenoviruses (AdV) are ubiquitous pathogens, and affect vertebrates, including humans, livestock and wild animals. They undergo genetic recombination and periodically emerge in the human population, often in geographically distinct patterns [1]. Human adenoviruses (HAdV) are comprised of ~60 types classified into seven species, A to G based on serology and DNA genome sequence [2, 3]. The genetic information of adenoviruses is encoded in a double-stranded linear DNA molecule. It is imported into the cell nucleus upon stepwise disassembly of incoming virions [4–7]. Adenovirus vectors are widely used in clinical gene therapy, mostly in cancer treatment and vaccination boost regimes [8], in part due to strong innate immunity reactions due to viral danger signals [9, 10]. Since the viral genome remains episomal throughout the infection cycle [11], this gives rise to a robust, but transient viral gene expression. In addition, AdV vectors are widely used in clinical oncolytic applications due to the ease of genetic engineering and biochemical modifications [12–15].

Most humans have been exposed to adenoviruses, and raise neutralizing antibodies and protective cytotoxic T cell responses [16–18]. In immune-competent individuals, AdVs cause only mild or no symptoms, with no observable long-term effects on health [19]. Persistent infections can occur [20] which may be caused by interferon based anti-viral defense mechanisms [21]. Adenoviruses can be live threatening to immune-compromised individuals [21–23]. The high seroprevalence and immunogenicity of adenoviruses represents a considerable drawback when using these viruses as vectors for gene therapy, as pre-existing immunity leads to virus immune-complexes which can lead to inflammatory reactions [13] and reduced treatment efficacy.

The AdV capsid is icosahedral, non-enveloped and consists of three major structural proteins fiber, penton base and hexon [24]. The facets of the capsid are composed of 240 copies of the homotrimeric hexon protein, while 12 copies of the fiber protein are attached to pentameric penton protein bases on each of the vertices. Each hexon trimer has a pseudo-hexagonal base, which allows for close packaging within the facet, and three tower domains that are exposed on the exterior surface of the virion. All three major capsid proteins are immunogenic [18, 25–28]. Occurrence of neutralizing antibodies against fiber protein in naturally infected individuals has been reported [18, 25, 29, 30].

Importantly, the bulk of functionally significant neutralizing antibodies are directed against the hexon protein [31–33]. The serotype-specific residues recognized by these antibodies are located in seven hyper-variable regions (HVRs 1-7) protruding from the outer side of the viral capsid as deduced by comprehensive alignment of hexon sequences [34–37]. Experiments with hexon chimeric viruses, in which the complete hexon, single or combinations of HVRs were replaced by those of another serotype, revealed escape of humoral immune detection [31, 32, 38–42]. Purified trimeric native hexon inhibited neutralization, but monomeric heat-denatured hexon did not, suggesting that neutralizing epitopes are complex and conformational but not linear [27, 34]. In contrast to these findings, other groups reported generation of neutralizing antibodies using small HVR peptides only [28, 43]. Non-neutralizing and cross-reactive hexon antibodies with common species- and genus-specific reactivity have been suggested to recognize sites that are accessible on the purified hexon but partially masked in the intact virion and outside of the HVRs [27, 44]. Such antibodies were characterized using complement fixation, immunodiffusion, immunoprecipitation and immunoblot assays [45–47].

Mouse adenoviruses (MAdV) represent an attractive model for studying viral and host factors involved in acute and persistent infections, testing oncolytic vectors in syngenic tumor models, and the development of anti-viral drugs [48–53]. Like HAdVs they belong to the genus of Mastadenoviruses. Reagents such as antibodies against MAdV are scarce. Here we devised and tested the use of minimal hexon fragments containing the HVRs 1-6 for production of specific hexon antibodies. Recombinant proteins generated in *E. coli* included hexon fragments derived from MAdV-1, −2, and HAdV-B3, −C5. Rabbit antibodies raised against MAdV-1, −2, and HAdV-3 fragments were tested in immunoblot assays, immunofluorescence, neutralization assays and EM for binding to denatured and native hexon. All three hexon antibodies recognized full-length hexon in Western immunoblots of lysates from infected cells, and two of three antibodies recognized native virus protein in immunofluorescence and transmission electron microscopy, but not in neutralization assays.

## Methods

### Cells and viruses

All cell lines including the human lung carcinoma cell line A549, the human cervical carcinoma cell line HeLa Ohio, the mouse colon carcinoma CMT93 and 3T6 fibroblast cells were grown in DMEM plus 8% FCS [15, 54, 55]. The cell lines were routinely screened for the absence of mycoplasma contamination. Human HAdV-C5 wt300 was obtained from T. Shenk [56]. All other human prototype viruses were kindly provided by the late T. Adrian (Medizinische Hochschule Hannover, Germany) and were verified by DNA restriction analysis [57] and in part by hexon sequence analysis (R. Hendrickx et al., manuscript in preparation). All human wild type viruses were amplified in A549 cells and viral titers were determined by plaque assay using 911 cells as described previously [54]. Recombinant E1/E3-deleted HAd-B3 and HAdV-C5 vectors expressing firefly luciferase were described previously [58]. MAdV-1, −2 and −3 were kind gifts of K. Spindler (University of Michigan, Ann Arbor, USA), S. Compton (Yale University School of Medicine, USA) and D. Krüger (Charité Campus Mitte, Berlin Germany), respectively, and were amplified in CMT93 or 3T6 cells and titered by qPCR (R. Hendrickx et al., manuscript in preparation). The replication-competent HAdV-B3-pIX-FS2A-eGFP contains an eGFP open reading frame (ORF) genetically fused to the downstream end of the pIX gene using an autocleavage FS2A sequence ([51] and L. Studer manuscript in preparation). Similarly, the replication-competent MAdV-1-E1A-FS2A-GL contains a *Gaussia* luciferase (GL) ORF genetically fused to the downstream end of the E1A gene (R. Hendrickx, manuscript in preparation). The MAdV2-∆E1A-eGFP contains an E1A exon 1 deletion which was replaced by an eGFP cassette (R. Hendrickx, manuscript in preparation).

### Construction of adenoviral hexon fragment expression vectors

For production of the four different hexon fragments the expression vector pGEX-6P-1 vector (GE Healthcare) was used. DNA fragments containing the hexon HVRs 1-6 were PCR-amplified using viral genomic DNA as template and inserted into the *Eco*RI and *Sal*I cloning sites of pGEX-6P-1. The oligonucleotide sequences (Microsynth AG, Switzerland) used for cloning are listed in STab 1. Hexon HVRs 1-6 protein sequences, and relative molecular sizes of the GST-hexon and final hexon fragments are summarized in Additional file 1: Figure S1 and Additional file 2: Table S1.

### Purification of adenoviral hexon protein fragments, production of rabbit polyclonal antibodies

Five grams of cell paste corresponding to a 4-l culture of *E.coli* BL21-CodonPlus(DE3)-RIPL cells, induced at 30 °C for 4 h with 0.3 mM IPTG, were resuspended in 25 ml of buffer T + 300 mM KCl (25 mM Tris-HCl, 10% glycerol, protease inhibitors (aprotinin, leupeptin, pepstatin A, PMSF), 0.5 mM EDTA, 1 mM dithiothreitol, 0.01% Nonidet-P40, pH 7.5). The cells were then disrupted by sonication and the lysate was clarified using ultracentrifugation (45 min, 4 °C, 100,000 × g).

The lysate was incubated with 1 ml of Glutathione-Sepharose beads (GE Healthcare) equilibrated with buffer T + 300 mM KCl for 1 h at 4 °C. The beads were then spun down and washed twice with 10 ml of T + 300 mM KCl and once with T + 50 mM KCl, followed by repeated protein elution with 1.5 ml of T + 100 mM KCl containing 30 mM glutathione. Fractions were analyzed by SDS-PAGE, pooled and treated with 100 units of PreScission Protease (GE Healthcare) for 22 h at 4 °C.

The solution was diluted with buffer 1xT until conductivity reached T + 50 mM KCl and was loaded on a 1 ml Mono Q column (GE Healthcare) equilibrated in T + 50 mM KCl. The protein was eluted using an 8 ml gradient of 50 – 500 mM KCl in buffer T.

Peak fractions were pooled, and loaded on a 5 ml Q5 Bio-Scale column (Bio-Rad) equilibrated in T + 50 mM KCl following adjustment of conductivity. The protein was eluted using a 100 ml gradient of 50 – 600 mM KCl in buffer 1xT. The elution times varied between different fragments but under these conditions, all of the fragments bound to the column, with HAdV-B3 eluting between 150 and 210 mM KCl, HAdV-C5 eluting between 270 and 350 mM KCl, MAdV-1 eluting between 90 and 130 mM KCl and MAdV-2 eluting between 60 and 110 mM KCl. Peak fractions were analyzed by SDS-PAGE, pooled and briefly incubated with a small amount of Glutathione-Sepharose beads to bind residual GST. The proteins were then concentrated using a 15 ml Vivaspin Turbo 15 concentration column with 10 kDa cut-off (Sartorius Stedim Biotech). All purification steps were performed at 4 °C and the final purified proteins were stored in buffer x at −80 °C until further use.

Purified hexon HVRs 1-6 fragments were used to immunize rabbits. Antibody development was carried out at BioGenes GmbH (Berlin, Germany). The animals were intramuscularly immunized using BioGenes’ adjuvant. The adjuvant was mixed 3:1 with the antigen. Final bleeds were done following four immunizations. The obtained sera were mixed with Thimerosal at 0.02% as preservative.

### Neutralization assay

The in vitro neutralization assay was performed as described in [59]. In brief, 2.5 × 10^4^ cells (3T6 for mouse viruses, A549 for human viruses) were seeded in 100 μl DMEM-FCS medium in 96 well plates and grown overnight at 37 °C. Serial five-fold dilutions or fixed dilutions of the hexon neutralizing antibody were prepared in 50 μl DMEM-FCS medium and mixed with 25 μl of either medium or increasing amounts of the hexon protein fragment. The mixes were incubated for 30 min at 37 °C. Subsequently, 25 μl of the reporter virus were added to the solutions and the mixes were incubated for another 30 min. Positive controls omitting the antibody and negative controls omitting the virus were included. The virus concentration was adjusted to a multiplicity of infection (MOI) of 5 in the case of the human viruses, whereas for MAdV-1-E1A-FS2A-GL 10^3^ genome equivalent virus particles (geq vp) per cell were used. The solutions were subsequently added to the cells and incubated for 24 h at 37 °C. For measuring the luminescence signal, the medium was aspirated and replaced with 40 μl of SteadyGLO lysis / substrate (Promega). The plates were incubated for 10 min at room temperature with orbital shaking. Then, 20 μl from each well was transferred to a Greiner LumiTrac plate and analyzed using a Tecan Plate reader with luminescence unit. When using MAdV-1-E1A-FS2A-GL, the renilla luciferase assay system (Promega) was used containing the coelenterazin substrate. All tests were performed in triplicates and repeated twice. The dog HAdV-C5 neutralizing serum was kindly provided by Anja Ehrhardt [60, 61]. The hyperimmune rabbit anti-HAdV-B3 was a kind gift from A. Kajon (Lovelace Respiratory Program, Albuquerque, USA).

### PAGE and western blot

For analysis of viral proteins derived from infected cells, cells were lysed in NETN (10 mM Tris pH 8.0, 200 mM NaCl, 1 mM EDTA, 0.5% NP40) complemented with protease inhibitors (Mini-Complete, Roche). Analyses of cleared cell lysates and hexon fragments from bacteria were performed by polyacrylamide gel electrophoresis [62] followed by Coomassie Blue or PageBlue™ (Thermo Scientific) staining, or by Western blotting of electrotransfered protein to Immobilon-P membranes as described previously [63]. Membranes were saturated in TBS-T containing 5% dry milk and incubated with primary antibodies including the rabbit anti-hexon fragment antibodies produced here, the rabbit anti-pIIIa antibodies (kindly provided by P. Hearing, School of Medicine, Stony Brook, USA) [64] (both 1:1000), and the mouse monoclonal anti-actin antibody (5A7 biorbyt UK), anti-GAPDH antibody (MA5-15738, Thermo Scientific), and anti-tubulin antibody (DM1A, Sigma) (all at 1 μg/ml). Incubation with secondary antibodies included HRP-conjugated donkey anti-rabbit IgG, sheep anti-mouse IgG (GE Healthcare, both 1:4000), and sheep anti-dog IgG (GE Healthcare) for 1 h. The immunoreactivity was determined using the Luminata Crescendo Western HRP substrate (Millipore) and scored using the ImageQuant LAS 4000 imager (GE Healthcare).

### Immunofluorescence imaging

1 × 10^4^ human A549 or mouse 3T6 cells were seeded in 50 μl of DMEM-FCS medium and were incubated at 37 °C for 3 h. The cells were then infected with virus using an MOI of 1 for HAdV-B3-pIX-FS2A-eGFP, or alternatively, 100 geq vp per cell for MAdV-1-ΔE1A-eGFP and MAdV-2-ΔE1A-eGFP. The replication-competent human virus gave rise to a strong GFP signals and cells were incubated for 1 day, whereas the mouse cells were incubated for 5 days, due to the weaker GFP signals produced by these viruses. The cells were fixed using 3% PFA for 15 min at room temperature. The cells were washed three times with PBS-N_3_ and quenched for 10 min at room temperature with PBS-N_3_ + 25 mM NH_4_Cl. The cells were washed again three times and blocked for 1 h at room temperature in PBS-N_3_ + 0.5% BSA. The cells were then incubated with primary antibodies diluted 1:500 in blocking buffer for one hour at 4 °C, washed three times and incubated with secondary antibodies for 1 h at room temperature. The cells were washed and 50 μl of PBS-N_3_ + DAPI was added to the wells at least 30 min prior to imaging. The cells were then imaged at a 20× magnification using a ImageXpress Micro XLS system.

### Immunogold-labelled electron microscopy imaging

Staining and analyses by transmission microscopy (TEM) was performed as described earlier [65]. In brief, 10 μl of concentrated CsCl-purified adenovirus solutions were pipetted onto glow discharged TEM mesh grids. Virus was left to adsorb for 1-2 min, excess buffer was dried using a filter paper and grids were washed four times for 1 min in 10 μl of PBS with 10% goat serum. Grids were then incubated in 10 μl of primary antibody diluted 1:50 in PBS with 1% goat serum for 30 min at room temperature. This was followed by additional washing four times for 1 min in PBS with 10% goat serum and then incubation with 10 μl of 1:50 diluted secondary colloidal 10 nm gold-conjugated antibodies for 30 min at room temperature. Grids were washed twice in PBS with 0.1% BSA, three times in PBS and twice with deionized water for 1 min each. Virus was counterstained with 3% uranyl acetate for 20-40 s and the excess staining solution was drained with filter paper. Grids were allowed to air dry and were then imaged using a TEM FEI Tecnai G2 electron microscope. The 9C12 antibody included as primary antibody was developed by Laurence Fayadat and Wiebe Olijve, and was obtained from Developmental Studies Hybridoma Bank developed under the auspices of the National Institute of Child Health and Human Development and maintained by the University of Iowa, Department of Biology, Iowa City, IA.

## Results

### Design and production of adenoviral hexon fragments

**Fig. 1:**
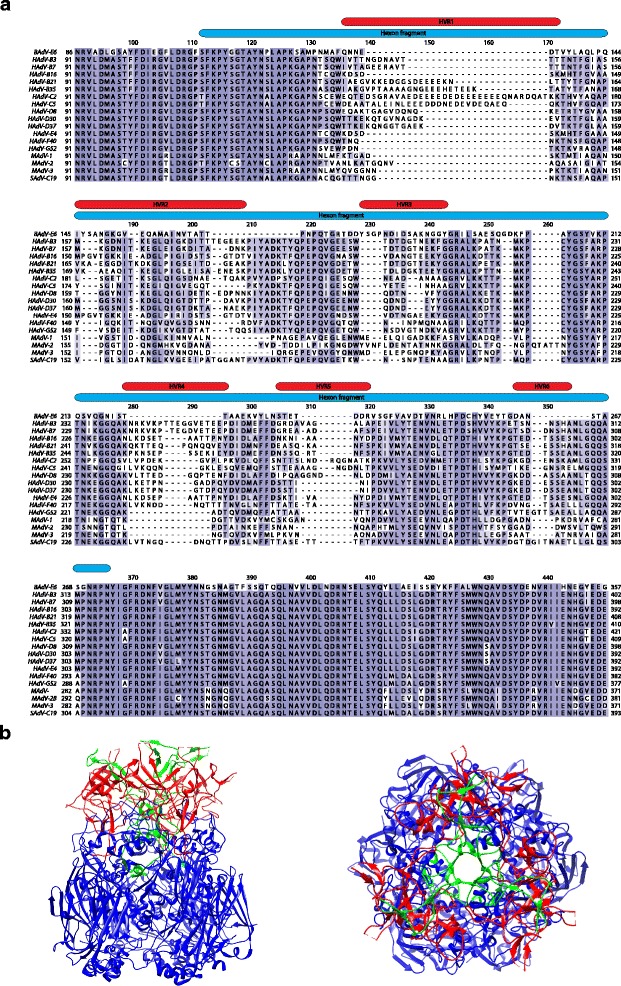
Alignment of partial adenovirus hexon sequences and hexon protein trimer structure. **a** Sequence alignment of 19 adenovirus hexon sequences containing HVRs 1-6. Protein sequences were obtained from GenBank and the alignment was performed using the MUSCLE algorithm. The color intensity indicates how highly an amino acid in a particular position is conserved amongst species. Highly conserved epitopes are highlighted in *dark blue* while variable epitopes are *white* or *light blue*. The HVRs 1-6 are highlighted by red bars and the cloned hexon fragments are highlighted by a *blue bar*. Hexon N- and C-terminal ends were omitted (for alignment of full-length hexon sequences see Additional file 1: Figure S1). **b** Graphical representation of the HAdV-C5 hexon protein trimer structure (PDB ID: 3TG7), side and top view. The HVRs 1-6 are highlighted in red and HVR 7 is highlighted in *green*

In the past, either purified virus or purified virus proteins were used for immunization to generate hexon-specific antibodies [44, 66]. Since in our hands, purified MAdVs yields were lower compared to HAdVs and purification of MAdV hexon protein from lysates of infected cells was not efficient (not shown), we decided to produce recombinant hexon fragments containing HVRs to induce specific antibodies. To this end, hexon protein sequences from recently described MAdV-2 [48] and −3 [49] were aligned to MAdV-1 and other known hexon sequences (Fig. 1a and Additional file 1: Figure S1). All seven HVRs characteristic for the sequence diversity across different adenoviral sequences [34–37] coincided with the highest variable stretches in the MAdV sequences.

Based on the previously published crystal structure of the trimeric form of the hexon protein (PDB ID: 3TG7) we then mapped these regions to the structural model of the HAdV-C5 hexon (Fig. 1b). We next designed adenoviral hexon fragments of about 20 kDa mass containing the HVRs 1-6, plus small parts of conserved regions at each end of the fragment to maximize the chances of preserving the secondary structure (Fig. 1a, Additional file 1: Figure S1 and Additional file 2: Table S1). In order to exclude sequences possibly containing common species- or genus-specific reactivity, and due to molecular size constraints, the HVR 7 was not included in the designed fragment as there is a highly extended region between the HVR 6 and 7.

Four HVRs 1-6 hexon fragments were generated including those of HAdV-B3, HAdV-C5, MAdV-1 and MAdV-2. The HAdV-C5 fragment was included as control to check for blocking capacities of such fragments (see below). The HAdV-B3 fragment and production of HAdV-B3 hexon antibodies was included to characterize HAdV-B3-derived vectors (L. Studer manuscript in preparation and [67]). All fragments were expressed in *E.coli* BL21-CodonPlus(DE3)-RIPL cells as N-terminal GST-fusion proteins containing a PreScission protease cleavage site, that allowed efficient enzymatic removal of the tag.

**Fig. 2:**
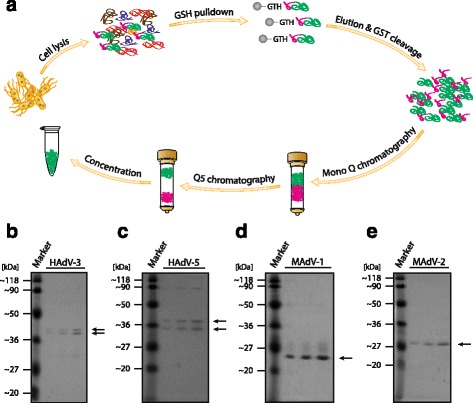
Purification of recombinant adenoviral hexon fragments. **a** Schematic representation of the purification process. Transformed *E.coli* BL21-CodonPlus(DE3)-RIPL cells were lysed to release cellular contents. The recombinant GST-fusion proteins were pulled down using Glutathione-Sepharose beads. After elution, the GST tag was cleaved and the hexon fragments were further purified using Mono Q and Q5 ion exchange chromatography. The resulting protein fractions were concentrated. **b**-**e** SDS-PAGE analyses of 1, 2 and 4 μl of purified HAdV-B3, HAdV-C5, MAdV-1 and MAdV-2 hexon fragments. Arrows indicate the bands of the purified proteins

A three-step purification procedure was employed to obtain highly pure hexon fragments (Fig. 2a). The first step was affinity purification using Glutathione-Sepharose beads. The GST-tagged protein bound to the affinity beads and could be separated from the majority of the contaminants. The GST-fusion hexon fragments as well as the final hexon fragments had a slightly slower mobility in SDS-PAGE than expected (Additional file 3: Figure S2A, Fig. 2b-e), which could be due to their relatively high negative charge (pI ~4-5) [68]. The eluted protein was treated with PreScission protease to cleave the GST-tag.

The second purification step consisted of an ion exchange separation on a Mono Q column which allowed to separate the cleaved hexon fragment from the GST tag. Apart from the HAdV-5 hexon fragment, the untagged hexon protein fragments were found in the flow through fraction, while the majority of the GST bound to the column (Additional file 2: Figure S2B). For the third purification step the Mono Q peak fractions were pooled and loaded on a second ion exchange Q5 column. All of the four fragments bound to the column and, after applying a KCl gradient, the hexon fragments were eluted revealing variable elution conditions (Additional file 3: Figure S2C). The purified protein fractions were briefly incubated with a small amount of Glutathione-Sepharose beads to bind residual GST and concentrate the hexon fragments. SDS-PAGE analysis revealed highly pure ≥90% hexon protein preparations (Fig. 2b-e). Apart from the MAdV-2 fragment, all other hexon fragments migrated as a double band, either after Q5 elution or after concentration. Mass-spectrometry analysis confirmed the identity of both bands, although the cause of the differential migration remained unclear. Possible reasons for heterogeneity are degradation, oxidation or post-translational modifications. It is difficult to ascertain the effect of this second band on immunisation. We believe that even if the other band were a slightly shortened product, most of the HVR epitopes should still be present and yield specific antibodies after immunisation. In the animal, professional antigen presenting cells process complex antigens into small peptide fragments and present these fragments on their surface which gives rise to the expansion of T-cells and eventually B-cells, giving rise to a systemic humoral immune response, including the production of immunoglobulins, which are of particular interest in this study.

In summary, our three-step purification procedure starting from a 4-l bacterial culture gave rise to highly pure hexon fragments with overall protein yields ranging from 600 to 850 μg. This material was further used for functional analysis and immunization of rabbits to raise polyclonal antibodies.

### The HAdV-C5 HVRs 1-6 hexon fragment blocks the neutralizing activity of a polyclonal antibody

**Fig. 3:**
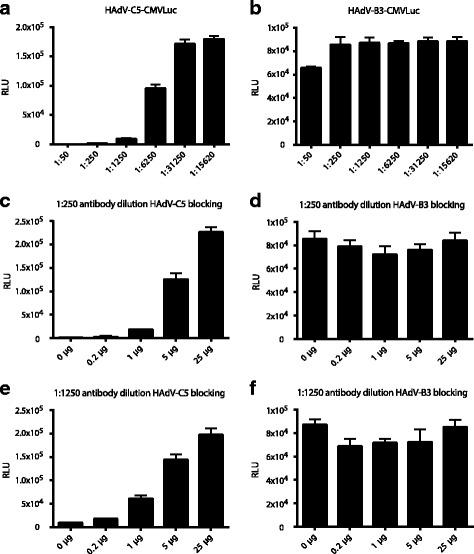
HAdV-C5 HVRs 1-6 hexon fragment efficiently blocks the neutralizing activity of an anti HAdV-C5 antibody. **a**, **b** Titration of the HAdV-C5 neutralizing serum. Five-fold serial dilutions of the neutralizing serum were pre-incubated with either HAdV-C5-CMVLuc or HAdV-B3-CMVLuc for 30 min before adding to human A549 cells. Luciferase activity was determined as relative light unit signal (RLU) in lysed cells 24 h post infection. **c**-**f** Blocking of HAdV-C5 neutralizing activity by the HAdV-C5 HVRs 1-6 hexon fragment. Two neutralizing antibody dilutions, 1:250 and 1:1250, which gave rise to <0.075% and ~5% residual HAdV-C5 activity, respectively, were used to repeat the neutralization assays in the presence of increasing amounts of hexon fragment. Blocking of neutralization could be seen in the homologous system using HAdV-C5-CMVLuc, but not in the heterologous system for HAdV-B3-CMVLuc-mediated expression

We tested whether the hexon fragment was able to bind HAdV-C5 neutralizing anti-hexon antibodies. If this were the case, it could improve the production of native hexon-recognizing antibodies. We evaluated the blocking activity of one of the generated fragments, the HAdV-C5 hexon fragment in a neutralization assay using a dog anti-HAdV-C5 neutralizing serum [60, 61]. As expected, the dog anti-HAdV-C5 serum stained various viral proteins including a protein corresponding in size to hexon (Additional file 4: Figure S3). We first tested the neutralization capacity of the dog serum against HAdV-C5-CMVLuc. When HAdV-C5-CMVLuc was pre-incubated with five-fold dilutions of the dog sera, the dog sera potently blocked expression mediated by HAdV-C5-CMVLuc in human A549 cells (Fig. 3a). The same serum did not block HAdV-B3-CMVLuc-mediated expression (Fig. 3b), confirming the neutralization specificity of the dog serum. A reduced luciferase activity effect of the dog serum at the 1:50 dilution against HAdV-B3-CMVLuc was obtained, but it was considered to be unspecific due to cytopathic effects observed on the cells, which resulted in a lower luciferase signal due to the reduced cell count. A possible reason for the cell cytopathic effect includes the presence of Thimerosal added as preservative to the sera, amounting at the 1:50 dilution to concentrations that had been reported to be toxic for human and mouse cells [69].

To test if the HAdV-C5 HVRs 1-6 hexon fragment acted as an antibody decoy, we repeated the neutralization assays in the presence of the hexon fragment. We used two neutralizing antibody dilutions, 1:250 and 1:1250, which previously had shown to gave rise to <0.075% and ~ 5% residual HAdV-C5 infection, respectively (Fig. 3a). Addition of increasing amounts of the hexon fragment increased the luciferase signal of HAdV-C5-CMVLuc, but not of HAdV-B3-CMVLuc-mediated expression (Fig. 3c-f). At the highest concentration of the HAdV-C5 HVRs 1-6 hexon fragment, blocking of the neutralizing activity was almost complete for both serum dilutions, suggesting that the dog serum did contain mainly hexon directed neutralizing antibodies. In summary, these data indicated that the HAdV-C5 HVRs 1-6 hexon fragment existed in a quasi native structure that sequestered and blocked virus-neutralizing antibodies.

### Hexon fragment antibodies reveal cross-reactivity and bind to full-length hexon protein in immunoblot assays

The purified HAdV-B3, MAdV-1 and MAdV-2 HVRs 1-6 hexon fragments were used to produce hyperimmune sera in rabbits. The resulting sera, hereafter referred to as HAdV-B3- / MAdV-1- / MAdV-2-hexon antibody (Ab), were tested in several assays to examine their properties.

To test the specificity of the generated antibodies, we performed immunoblot assays using lysates from cells infected with thirteen human and three mouse viruses. Human HeLa cells were infected with HAdVs representing four species and included HAdV-B3, HAdV-B7, HAdV-B16 and HAdV-B21 of the B1 subspecies, HAdV-B11, HAdV-B14, HAdV-B34 and HAdV-B35 of the B2 subspecies, HAdV-D8, HAdV-D30 and HAdV-D37 of the D species, and HAdV-C5 and HAdV-E4 from the C and E species, respectively. Mouse CMT93 cells were infected with all three known MAdVs. In addition to the hexon antibodies, all blots were also immunostained using a HAdV cross-reactive anti-pIIIa antibody [64] to check for efficient infection, and an actin antibody to check for sample loading. pIIIa is a conserved structural protein contributing to capsid structure and stability. In our experiments, it serves as loading control and a marker for production of late transcribed genes, including hexon.

**Fig. 4:**
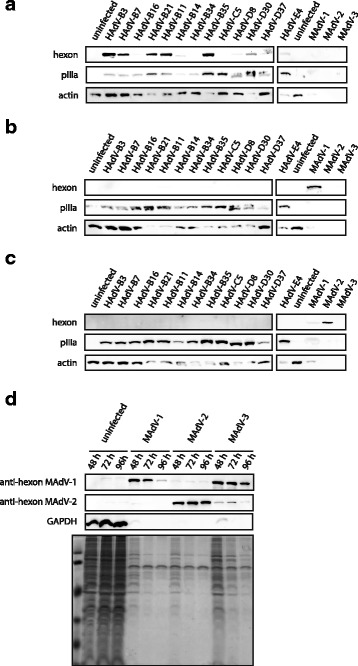
Antibodies raised against hexon fragments reveal variable cross-reactivity against hexon proteins in Western immunoblotting. Lysates from HeLa cells infected with thirteen HAdVs representing the four species B, C, D and E and CMT93 lysates with all three different MAdVs were analyzed. Uninfected HeLa or CMT93 cells were included as negative controls, respectively. The blots were stained with the HAdV-B3 hexon Ab (**a**), the MAdV-1 hexon Ab (**b**), or the MAdV-2 hexon Ab (**c**). In addition, all blots were immunostained using a (HAdV) cross-reactive anti-pIIIa antibody to check for efficient infection, and an actin antibody for loading control. It was noticed that the actin signal in mouse cells infected with MAdVs strongly faded in the course of infection. **d** A second set of CMT93 cells infected with all three MAdVs at about five-fold higher virus input were harvested 48, 72 and 96 h post infection. The blot was immunostained using the MAdV-1 and MAdV-2 hexon Ab and GAPDH loading control antibody. The same lysates were stained for protein content using PageBlue. Marker proteins of 118, 85, 48, 34, 26 and 20 kDa were included in the first lane

The HAdV-B3 hexon Ab strongly bound to the full-length HAdV-B3 hexon, and also cross-reacted strongly with most of the other hexons of the B1 and B2 subspecies viruses, and with the hexons of the D serotypes included here (Fig. 4a). Of note, weak signals of HAdV-B14 and B34 of B2 subspecies were paralleled by relative weak pIIIa signals, whereas pIIIa signals of all D serotypes were rather robust. Hexons from HAdV-C5 and HAdV-E4, as well as HAdV-B16 revealed weak binding and cross-reactivity.

When testing the MAdV-1 hexon Ab in a first experiment with mouse CMT93 lysates harvested 72 h post infection, it bound strongly to the MAdV-1 full-length hexon, and weakly to the MAdV-3 hexon, which are highly related in sequence [48] (Fig. 4b). In addition, the MAdV-1 hexon Ab exhibited low affinity towards the HAdV-B11 and HAdV-B35 hexon. The MAdV-2 hexon Ab displayed low cross-reactivity to MAdV-1 hexon, but strongly bound to the MAdV-2 full length hexon (Fig. 4c). It was also noticed that the cross-reactive anti-pIIIa antibody did not efficiently recognize pIIIa from MAdVs which could be explained by the low sequence similarity to human adenoviruses in certain segments of the protein [20]. Further, in contrast to cell lysates obtained from HAdV infections, the actin signal in mouse cells infected with MAdVs faded in the course of infection.

We repeated the infection experiment using an about 5-fold higher virus input followed by harvesting cell lysates 48, 72 and 96 h post infection. In addition to immunoblotting analyses, the cell lysates were also analyzed for protein levels by PageBlue staining (Fig. 4d). Surprisingly, all MAdV-infected lysates revealed a robust reduction in cell protein contents at all three time points analyzed, when compared to uninfected cells. When immunostaining for GAPDH, weak signals were seen for MAdV-1 and -3 only at 48 h post infection Staining for actin and tubulin showed similar results (not shown), in agreement with the reduced levels of cellular proteins. In this experiment with higher virus input, the MAdV-1 hexon Ab bound to similar extents to MAdV-1 and MAdV-3 full-length hexon proteins. The MAdV-2 hexon Ab displayed low cross-reactivity to MAdV-1 as well as to MAdV-3 hexon protein.

In summary, all three sera produced against hexon HVRs 1-6 fragments revealed binding to homologous full-length hexon protein demonstrating variable degrees of cross-reactivity to heterologous hexons when tested for binding in immunoblot assays representing mostly non-native forms of hexons.

### HAdV-B3- and MAdV-2- but not MAdV-1-hexon fragment antibodies display hexon signals in IF analysis of infected cells and in immuno-EM of intact virions

**Fig. 5:**
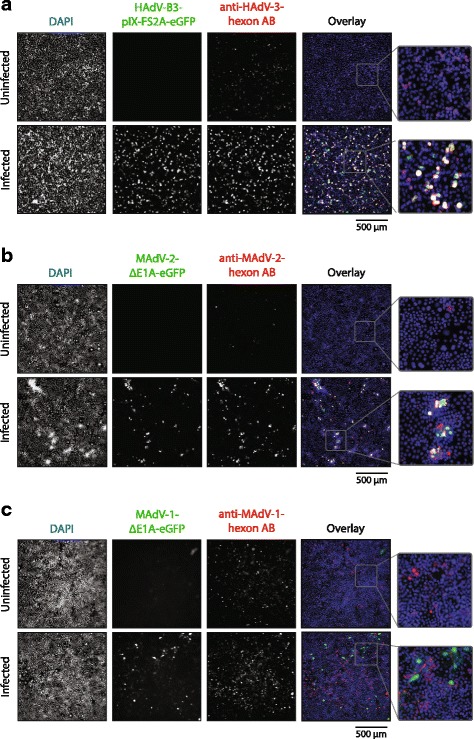
Use of hexon fragment antibodies in immunofluorescence analysis of GFP-reporter virus infected cells. **a** Human A549 cells were infected with replication-competent HAdV-3-pIX-FS2A-eGFP at an MOI of 1 and cells were fixed and stained one day post infection with the HAdV-B3 hexon Ab. Co-localization of a HAdV-B3 hexon specific signal with the GFP signal was observed in the overlays. **b** Mouse 3T6 cells were infected with 100 geq vp of MAdV-2-ΔE1A-eGFP and cells were fixed and stained five days post infection with the MAdV-2 hexon Ab. Co-localization of the MAdV-2 hexon signal with GFP was observed in channel overlays. **c** Mouse 3T6 cells were infected with 100 geq vp of MAdV-1-ΔE1A-eGFP and cells were fixed and stained five days post infection with the MAdV-1 hexon Ab. No co-localization of GFP and hexon signal was observed. DAPI staining is highlighted in *blue*, the GFP signal is highlighted in green and the hexon antibody signal is highlighted in *red*

Immunofluorescence (IF) microscopy is widely used to study the infectious life cycle of viruses. To investigate whether the hexon fragment antibodies were suitable for use in IF, human and mouse cells were infected with eGFP expressing reporter viruses followed by analysis for eGPF and hexon signals. When human A549 cells were infected with replication-competent HAdV-B3-pIX-FS2A-eGFP we were able to observe a strong hexon signal that overlapped with the GFP signals (Fig. 5a). Of note, uninfected cells revealed a weak background signal when using the HAdV-B3 hexon Ab. Similarly, when mouse 3T6 cells were infected with recombinant MAdV-2-∆E1A-eGFP, hexon and GFP signals coincided to a large degree, whereas uninfected cells revealed a very low background staining with the MAdV-2 hexon Ab (Fig. 5b). In contrast, when 3T6 cells infected with MAdV-1-∆E1A-eGFP virus were stained with the MAdV-1 hexon Ab, a high background was observed with signals that did not overlap with the GFP signals (Fig. 5c).

**Fig. 6:**
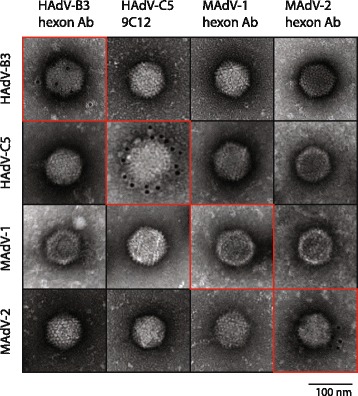
Transmission electron microscope analysis of immunogold-labeled viral capsids. Purified HAdV-B3, HAdV-C5, MAdV-1 and MAdV-2 were first incubated with the indicated rabbit hexon antibodies for HAdV-B3, MAdV-1, MAdV-2, or the monoclonal mouse 9C12 antibody specific for HAdV-C5, respectively. Following staining with colloidal 10 nm gold-labeled anti-rabbit or anti-mouse antibodies, uranyl-acetate staining was performed. TEM images were acquired at 135 000× magnification

These findings were corroborated when the same antibodies were used for staining of CsCl-purified viruses by high-resolution immunogold EM. For this, wild type HAdV-B3, HAdV-C5, MAdV-1 and -2 were first incubated with the three hexon-fragment antibodies or with the mouse monoclonal 9C12 antibody specifically binding to HAdV-C5 [59], followed by incubation with gold-labeled secondary antibodies (Fig. 6). When staining HAdV-C5 with the 9C12 antibody, we observed multiple gold grains on the HAdV-5 capsid surface. This staining was specific, since none of the other virus capsids were bound by the 9C12 antibody. Gold grains were also seen when HAdV-B3 was incubated with the HAdV-B3 hexon Ab, or MAdV-2 was incubated with the MAdV-2 hexon Ab, albeit at lower numbers than for 9C12. In contrast, in the case of MAdV-1 we were not able to detect immune-gold particles on the intact viral capsids. In summary, our IF and EM results suggest that HAdV-B3 and MAdV-2 hexon Abs recognized in addition to non-native also native forms of hexon, whereas the MAdV-1 hexon HVRs 1-6 antibodies recognized only non-native hexon forms.

### HAdV-B3- and MAdV-1-hexon Abs exhibit no neutralizing activity on virus infection

**Fig. 7:**
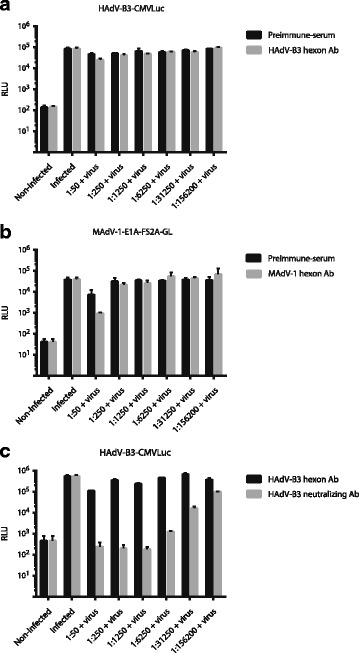
HAdV-B3 and MAdV-1 hexon Abs do not reveal neutralizing activity. **a** Five-fold serial dilutions of preimmune serum or HAdV-B3 hexon Ab were pre-incubated with HAdV-B3-CMVLuc for 30 min before adding to human A549 cells. **b** Five-fold serial dilutions of preimmune serum or MAdV-1 hexon Ab were pre-incubated with MAdV-1-E1A-FS2A-GL for 30 min before adding to mouse CMT93 cells. For both viruses, luciferase activity was measured as RLU in lysed cells 24 h post infection. **c** Comparison of the neutralizing activities of the HAdV-3 hexon Ab with a neutralizing rabbit anti HAdV-B3 serum. Dilutions and experimental set-up were as described above

Since two of our hexon Abs recognized native hexon forms, we tested if they also had neutralizing activity. To test this we repeated the neutralizing assays using HAdV-B3-CMV-Luc and MAdV-1-E1A-FS2A-GL for infection of human and mouse cells, respectively. When compared to the pre-immune sera, neither the HAdV-B3 hexon Ab (Fig. 7a) nor the MAdV-1 hexon Ab (Fig. 7b) revealed any neutralizing activity. As seen previously with the dog anti-HAdV-C5 serum, a small cytopathic effect induced reduction of luciferase expression at the 1:50 dilution of both sera. When including as positive control a hyperimmune rabbit anti-HAdV-B3 serum, HAdV-B3-CMV-Luc-mediated luciferase expression was strongly blocked (Fig. 7c). The neutralizing activity of the MAdV-2 hexon Ab could not be tested here, due to the lack of a MAdV-2 luciferase reporter virus.

## Discussion

Standard procedures to generate adenovirus hexon-specific antibodies include preparations of purified virions, or viral proteins for the purpose of immunization [44, 66]. However, not all adenoviruses can be amplified efficiently in cell culture. For instance the two HAdVs belonging to species F (HAdV-F40 and −41) are notoriously difficult to grow to high titers [70]. When trying to isolate MAdV-1 and-2 hexon protein from infected cell cultures using standard protocols [71] we failed to obtain purified proteins. Since hexon is a large protein of 108 kDa that requires a viral chaperone activity [72], production in *E.coli* was found to be inefficient (unpublished data).

We decided to generate a small hexon subfragment consisting of about one fifth of the full-length size, but still containing six of the seven HVRs (Fig. 1a, Additional file 1: Figure S1 and Additional file 2: Table S1). HVR 7 was omitted since inclusion of this region increases the fragment size by about two-fold. In addition, neutralization profile analysis of hexon chimeric HAdV-C5 containing either HVRs 1-7 or only HVRs 1-6 of HAdV-C2 as well as epitope mapping of chimpanzee Ad68 hexon using a panel of neutralizing antibodies had suggested that most reactivity was contained in the HVRs 1-6 sequence [27, 38]. Similar data were also reported with HAdV-B3 and -B7 [73, 74]. In contrast, mapping of the epitope recognized by the mouse monoclonal 9C12 anti-HAdV-C5 hexon antibody suggested also a contribution of HVR 7 to binding of this neutralizing hexon antibody [59, 75].

Following efficient expression in bacteria, our three-step purification procedure of the hexon HVRs 1-6 fragments using standard biochemical methods resulted in >90% pure protein preparations based on SDS-PAGE analysis, with sufficient quantities for immunization and production of polyclonal antibodies in rabbits (Fig. 2). It was somewhat surprising to find that the HAdV-C5 fragment was almost completely blocking a dog-anti HAdV-C5 neutralizing activity although the fragment consisted only of HVRs 1-6 (Fig. 3). Hexon fragment proteins consisting of HVRs 1-7 have been used for similar blocking experiments [42], as well as a HVR 5-derived small peptide of HAdV-C5 [28]. However, blocking activity by peptides may be rather exceptional, since it has been estimated that only a small fraction of <10% of the antibodies directed against native antigens bind to linear epitopes [76]. In the case of HAdV-D48 hexon, neutralizing antibodies did not recognize linear peptides [34]. Whether all of the four hexon fragments are capable of blocking neutralizing antibodies remains to be determined once the recombinant reporter viruses are available.

Hexon antigenic determinants and antibodies raised against hexon will not only be influenced by the nature of antigen used for immunization, e.g., purified virus, or hexon or partial fragment, but also by the immunization procedure which can include use of alum precipitation, adjuvants, and terminal boost with virus only. Thus, generation of mouse monoclonal antibodies resulted in cross-reactive genus-, species- or type-specific antibodies [44, 66]. Type-specific and neutralizing epitopes have been suggested to be conformation dependent [27, 34], whereas non-neutralizing and cross-reactive hexon antibodies with common species- and genus-specific reactivity have been suggested to recognize sites that are accessible on the purified hexon but partially masked in the intact virion and outside of the HVRs [27, 44, 45].

A variable degree of cross-reactivity binding to heterologous hexons was observed when testing our three hexon Abs for binding in immunoblot assays, representing binding to mostly non-native forms of hexons. In the case of the HAdV-B3 hexon Ab, extensive cross-reactivity with most of the other tested hexons of the B1 and B2 subspecies viruses, and with the hexons of the D types were noticed (Fig. 4a). The observed cross-reactivity between species B and D is confirmed by phylogenetic tree analyses data of conserved and variable hexon regions, revealing that the B and D species viruses are more closely related than the B and C species viruses [77]. The reduced cross-reactivity to hexon of HAdV-B16 is in line with the previously noted clustering of the HAdV-B16 hexon sequence to the HAdV-E4 sequence, suggested to have resulted from a recombination between sequences of these viruses [77, 78]. Since the MAdVs clade is rather distant from the other Mastadenovirus clades [48], it is not surprising to find lower levels of cross-reactivity of the MAdV-1 hexon antibodies and almost no cross-reactivity with the MAdV-2 hexon antibodies (Fig. 4b, c). The MAdV-1 hexon directed antibodies bound stronger to the MAdV-3 hexon than to the MAdV-2 hexon, reflecting the closer ancestor relationship of MAdV-1 and -3 compared to MAdV-2 [48].

When used for detection of hexon proteins in IF of infected cells and for EM of purified virus, the HAdV-B3 and MAdV-2 hexon antibodies, but not the MAdV-1 antibody bound native hexon proteins (Figs. 5 and 6). However, when used for virus neutralizing assays, neither the HAdV-B3 nor the MAdV-1 antibodies revealed any blocking effect (Fig. 7), suggesting that the MAdV-1 antibody exclusively detects nonnative and denatured epitopes, whereas the HAdV-B3 antibody recognizes native hexon epitopes to some degree, but not those epitopes contained on trimeric hexons leading to neutralization.

Our results with the HAdV-B3 hexon antibody is in contrast to a study by Yuan et al. which generated HAdV-B3 neutralizing antibodies immunizing with various short HVR peptides bound to KLH carrier protein [43]. However, the Yuan et al. study did not include other virus serotypes to confirm the specificity. Previous studies have shown that not all hexon binding antibodies exert neutralizing activity, but those that do bind with high affinity and function by rapid, single-hit kinetics, acting at a post-entry and TRIM21-dependent step [18, 27, 79, 80]. Non-neutralizing antibodies were found to bind with lower avidity to native hexon than neutralizing antibodies [27], which could explain why two of our three hexon antibodies still bound to intact virus in EM analysis, despite their lack of neutralization.

## Conclusions

The procedure described here can be applied to generate monoclonal or polyclonal antibodies against adenoviruses that are difficult to grow in cell culture. Such antibodies can greatly speed up the process of optimizing the cultivation and purification conditions of the virus, and enhance studies of the infection cycle and serve as diagnostic tools in tissue analyses. Purified hexon fragments can be used in a variety of biochemical assays apart from immunizing animals, for instance blocking neutralization assays, pull-down assays or competition assays. The method described here is cost effective and yields milligram quantities of recombinant soluble hexon protein. It extends traditional methods, and improves adenoviral research and diagnostics.

## Additional files

Additional file 1: Figure S1. Protein sequence alignment of 19 full-length adenoviral hexon proteins. Protein sequences were obtained from GenBank and the alignment was performed using the MUSCLE algorithm. The HVRs1-6 are highlighted by red bars, HVR7 by a green bar and the cloned hexon fragment is highlighted by a blue bar. (PDF 795 kb)

Additional file 2: Table S1. Hexon fragment data and oligonucleotides used for generation of expression constructs. (DOC 45 kb)

Additional file 3: Figure S2. Adenoviral HVRs 1-6 hexon fragment purification. SDS-PAGE analyses of HVRs1-6 hexon fragments of HAdV-B3 HAdV-C5, MAdV-1 and -2 were performed after GSH affinity purification (A), Mono Q ion-exchange purification (B), and Q5 ion-exchange purification (C). The Flow fraction represents proteins that did not bind to the chromatography matrix, the Wash fraction represents proteins washed out during the washing step of the purification, and the Beads fraction represents proteins that were eluted from the matrix by SDS boiling. Elution from the Q5 ion-exchange column was performed applying a slow KCl gradient of 50-600 mM. The hexon fragments were eluted at different KCl concentrations, including 150-210 mM KCl for the HAdV-B3 fragment, 270-350 mM KCl for the HAdV-C5 fragment, 90-130 mM KCl for the MAdV-1 ragment and 60-110 mM KCl for the MAdV-2 fragment. (PDF 2150 kb)

Additional file 4: Figure S3. Immunostaining of hexon by the neutralizing dog anti-HAdV-C5 serum. Lysates from uninfected and infected Hela cells were analyzed by Western immunoblot using the polyclonal dog anti-HAdV-C5 serum. Several viral proteins including a protein corresponding in relative size to hexon (108 kDa) were detected. (PDF 346 kb)

## Abbreviations

Ab: Antibody
AdV: Adenoviruses
geq vp: Genome equivalent virus particles
GL: *Gaussia* luciferase
HAdV: Human adenoviruses
HAdV-B3: Human adenovirus 3
HVRs: Hyper-variable regions
IF: Immunofluorescence
MAdV: Mouse adenoviruses
MOI: Multiplicity of infection
ORF: Open reading frame
RLU: Relative light unit signal
TEM: Transmission microscopy
