# Supplementary figures and images for: Small-size recombinant adenoviral hexon protein fragments for the production of virus-type specific antibodies
Source: Virol J. 2017 Aug 18;14:158. doi: 10.1186/s12985-017-0822-5 (PMC5563037; doi:10.1186/s12985-017-0822-5)

A

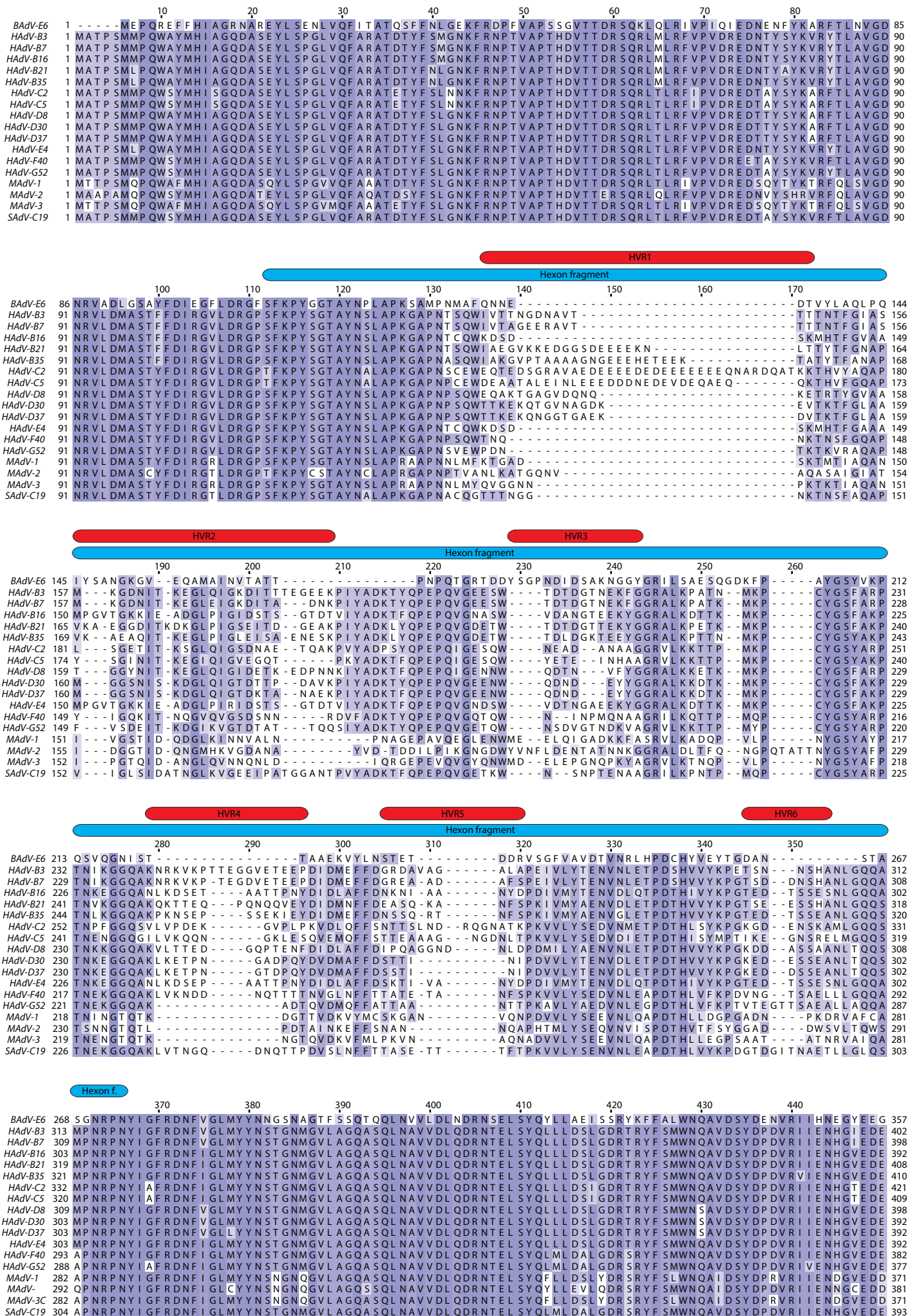

Supplement: Supplementary file 1 — Protein sequence alignment of 19 full-length adenoviral hexon proteins. Protein sequences were obtained from GenBank and the alignment was performed using the MUSCLE algorithm. The HVRs1-6 are highlighted by red bars, HVR7 by a green bar and the cloned hexon fragment is highlighted by a blue bar. (PDF 795 kb) [file 12985_2017_822_MOESM1_ESM.pdf]

A

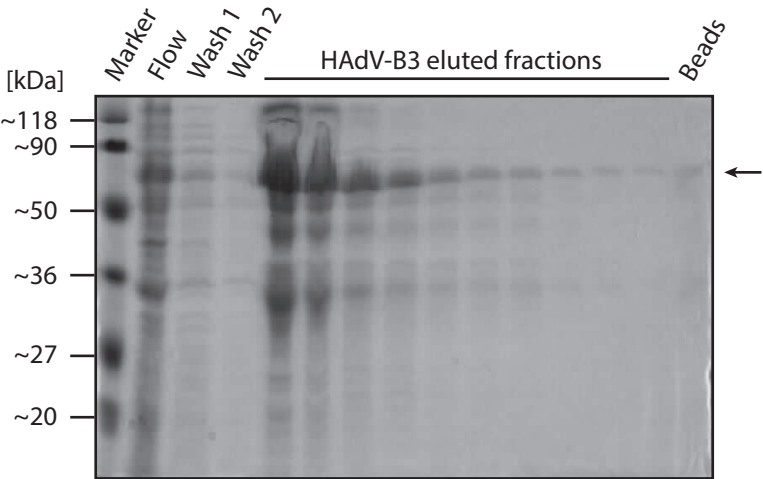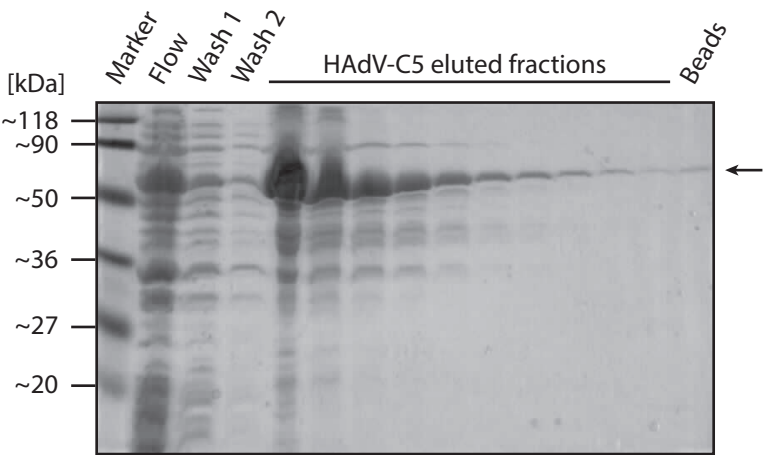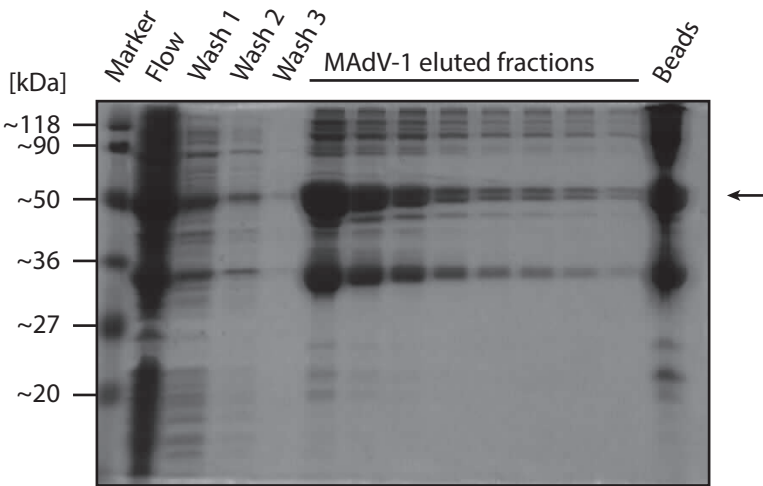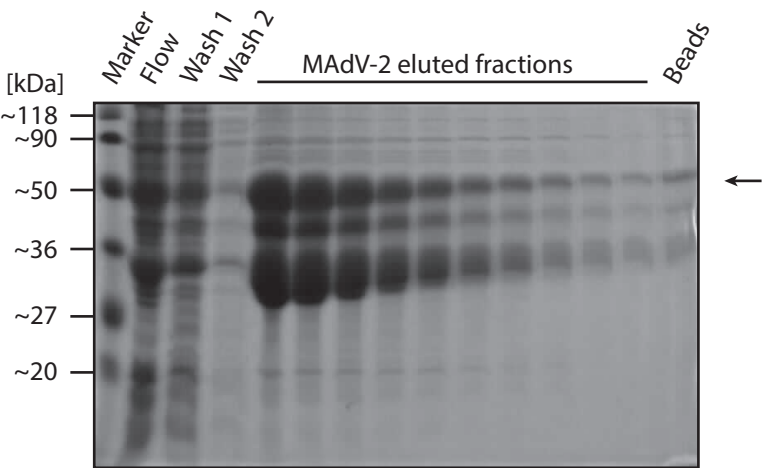

B

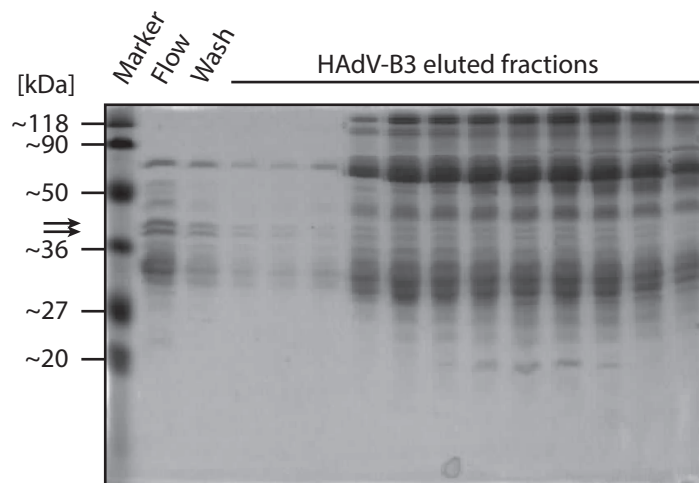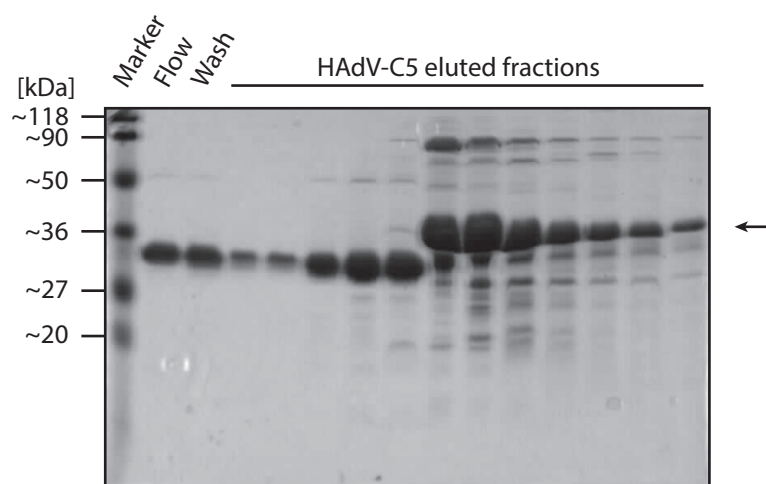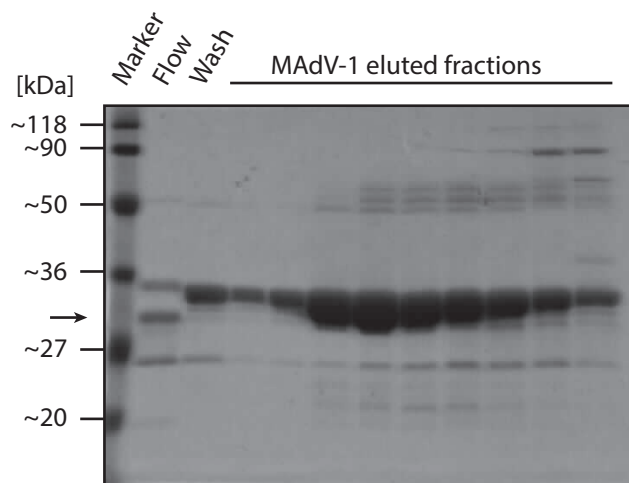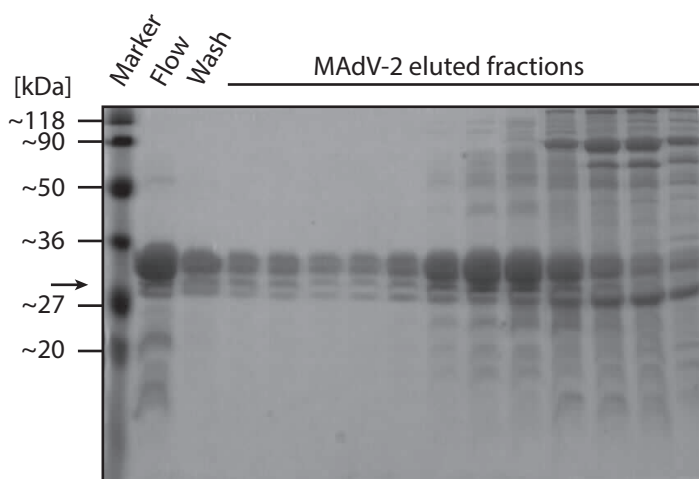

C

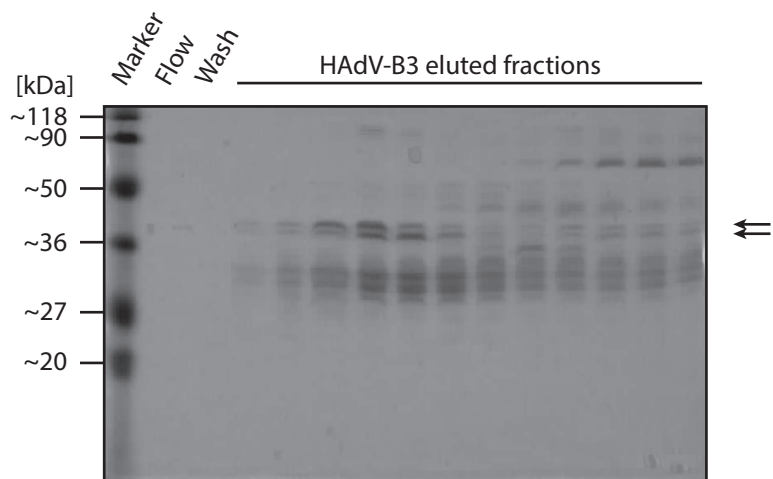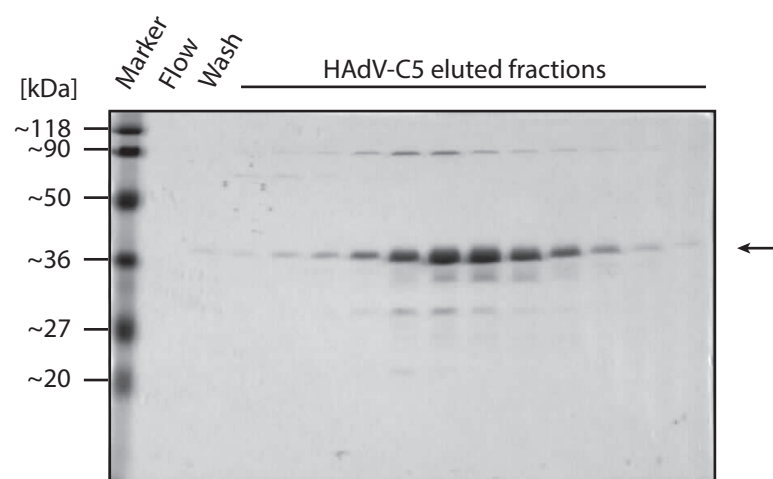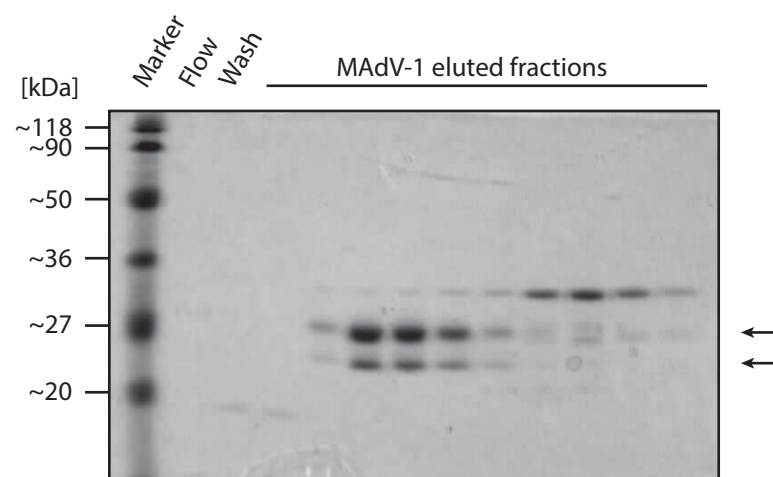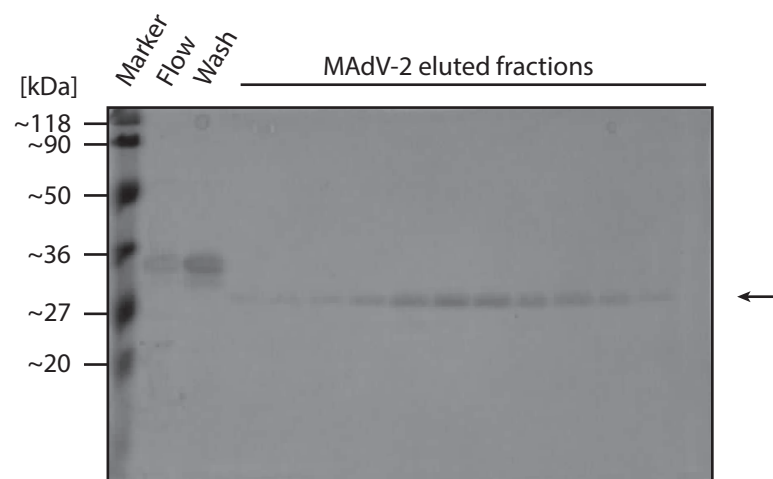

Supplement: Supplementary file 3 — Adenoviral HVRs 1-6 hexon fragment purification. SDS-PAGE analyses of HVRs1-6 hexon fragments of HAdV-B3 HAdV-C5, MAdV-1 and -2 were performed after GSH affinity purification (A), Mono Q ion-exchange purification (B), and Q5 ion-exchange purification (C). The Flow fraction represents proteins that did not bind to the chromatography matrix, the Wash fraction represents proteins washed out during the washing step of the purification, and the Beads fraction represents proteins that were eluted from the matrix by SDS boiling. Elution from the Q5 ion-exchange column was performed applying a slow KCl gradient of 50-600 mM. The hexon fragments were eluted at different KCl concentrations, including 150-210 mM KCl for the HAdV-B3 fragment, 270-350 mM KCl for the HAdV-C5 fragment, 90-130 mM KCl for the MAdV-1 ragment and 60-110 mM KCl for the MAdV-2 fragment. (PDF 2150 kb) [file 12985_2017_822_MOESM3_ESM.pdf]

A

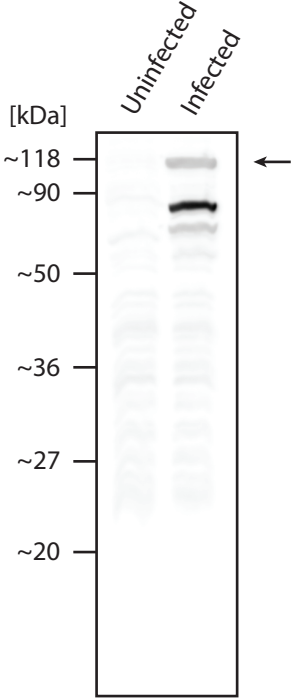

Supplement: Supplementary file 4 — Immunostaining of hexon by the neutralizing dog anti-HAdV-C5 serum. Lysates from uninfected and infected Hela cells were analyzed by Western immunoblot using the polyclonal dog anti-HAdV-C5 serum. Several viral proteins including a protein corresponding in relative size to hexon (108 kDa) were detected. (PDF 346 kb) [file 12985_2017_822_MOESM4_ESM.pdf]
