# Supplementary material for: Small-size recombinant adenoviral hexon protein fragments for the production of virus-type specific antibodies
Source: Virol J. 2017 Aug 18;14:158. doi: 10.1186/s12985-017-0822-5 (PMC5563037; doi:10.1186/s12985-017-0822-5)
Supplement: Supplementary file 2 — Hexon fragment data and oligonucleotides used for generation of expression constructs. (DOC 45 kb) [file 12985_2017_822_MOESM2_ESM.doc]

Additional file Table S1. Hexon fragment data and oligonucleotides used for generation of expression constructs

| **HVRs 1-6 construct** | **HVRs protein sequence** | **Mr**  **final**  **protein** | **Mr**  **GST-protein** | **Oligo**  **Name** | **Oligo-Sequence (5’-3’)** |
| --- | --- | --- | --- | --- | --- |
| HAdV-B3 | 112 -316 | 22 kDa | 48 kDa | pR2570 | ATTGGAATTCACTTTTAAGCCCTACTCTGGC |
|  |  |  |  | pR2571 | AGCTGTCGACTTAAGGCCTGTTGGGCATAGATTG |
| HAdV-C5 | 112 -324 | 23 kDa | 49 kDa | pR2576 | GCAAGAATTCAGCTTTAAACCCTACTCAGGAACGG |
|  |  |  |  | pR2577 | ACCAGCATGCAGCTTTAAACCCTACTCAGGAACGG |
| MAdV-1 | 112 -286 | 19 kDa | 45 kDa | pR2661 | GTTAGAATTCAGCTTCAAGCCATATTCCG |
|  |  |  |  | pR2662 | TGGTGTCGACTTAGGTCTGTTAGGCATGGCTTG |
| MAdV-2 | 112 -296 | 20 kDa | 46 kDa | pR2686 | GTAAGAATTCACGTTCAAACCCTACTGCAGC |
|  |  |  |  | pR2687 | TGTTGTCGACTTAGGGTCGGTTGGGCTGTGACCAC |
